# Effects and mechanisms of a microcurrent dressing on skin wound healing: a review

**DOI:** 10.1186/2054-9369-1-24

**Published:** 2014-11-24

**Authors:** Chao Yu, Zong-Qian Hu, Rui-Yun Peng

**Affiliations:** Beijing Institute of Radiation Medicine, Beijing, 100850 China

**Keywords:** Microcurrent dressing, Electric stimulation, Skin wound healing

## Abstract

The variety of wound types has resulted in a wide range of wound dressings, with new products frequently being introduced to target different aspects of the wound healing process. The ideal wound dressing should achieve rapid healing at a reasonable cost, with minimal inconvenience to the patient. Microcurrent dressing, a novel wound dressing with inherent electric activity, can generate low-level microcurrents at the device-wound contact surface in the presence of moisture and can provide an advanced wound healing solution for managing wounds. This article offers a review of the effects and mechanisms of the microcurrent dressing on the healing of skin wounds.

## Introduction

Skin, the natural protective barrier for the body, plays an important part in defense against foreign bodies and pathogens, helps retain water and electrolytes, and maintains homeostasis. It is essential to maintain skin integrity. Surgical wounds, burns, and a variety of chronic skin ulcers damage the skin and can compromise the skin’s protective nature. Despite recent advances in the understanding of the biology of healing, an unmet need remains in handling the clinical problem of skin wounds, particularly persistent skin ulcers. To reestablish the skin’s barrier function, it is necessary to apply a proper wound dressing as a temporary substitute to damaged skin. In addition to defending against foreign bodies and pathogens, wound dressings should replicate skin characteristics to promote the proliferation and migration of fibroblasts and keratinocytes and enhance re-epithelialization, leading to proper and rapid healing with minimal scar formation.

Wound dressings have changed significantly over time. The development started with the use of natural materials to simply cover the wounds and has progressed to cutting-edge materials that can be specially made to exhibit various extraordinary functions. A microcurrent dressing (MCD) is a unique wound dressing with wireless microcurrent technology that provides an advanced wound healing solution. In the presence of moisture, low-level microcurrents are generated at the device-wound contact surface. These reactions occur without an external power source or other accessories; it is a wireless, conformable and portable device.

### Microcurrent dressing

The main function of MCD is to provide electric stimulation (ES) therapy, which is broadly defined as the application of an electric current through electrodes placed on the skin, near or directly within the wound. It has been established that the human epidermis acts as a battery, and when its integrity is broken, it generates an electric field on the immediate wound edge [1, 2]. Based on the observed endogenous electrical properties, it has been hypothesized that the external application of electrical current can be employed to assist in the healing of skin wounds [3]. ES offers an external wound treatment that has been shown to have a positive effect on wound healing in many clinical studies [4–7].

ES therapy has been reported for decades as a therapeutic method to aid and promote wound healing. As early as 1969, in vivo preclinical studies on ES therapy were conducted, followed by numerous animal and clinical studies to support its application. Studies on cutaneous wound healing in animal models became more prevalent in the 1990s. In 2002, the American Food and Drug Administration granted premarket approval for the clinical use of ES devices to treat certain chronic wounds (e.g., diabetic, pressure (stage III or IV), stasis, and arterial ulcers) that had failed standard wound therapies [8]. Recent advances in research of the electrical phenomena in the skin have aroused an interest in this modality [2, 9].

**Figure 1:**
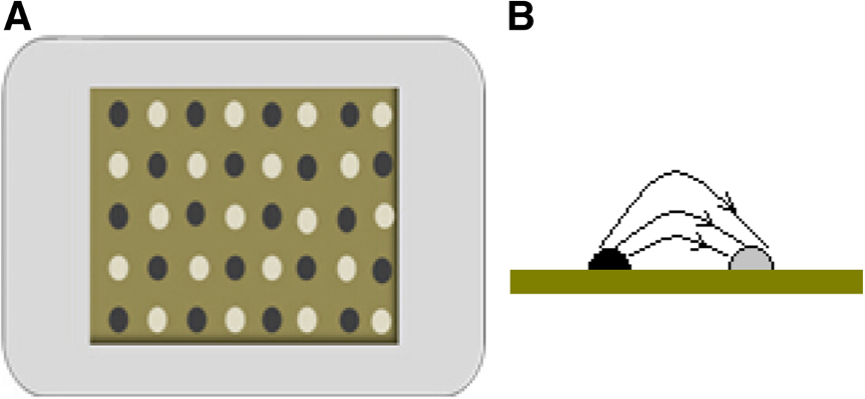
**The sketch of a microcurrent dressing. (A)** The dots of different color stand for dissimilar reservoirs. One type of reservoir includes oxidizing agents, and the other includes reducing agents. **(B)** One of the coupled dissimilar reservoirs. In the presence of moisture, redox reactions will occur, and currents will thus be produced. Accordingly, a field of multiple currents will be produced across a surface of a substrate.

There are several types of ES devices have been used to treat wounds. One example is a portable electric dressing device that integrates low-level ES into wound dressings [10]. Generally, electric dressing devices can be divided into two types: the wired electric dressing and the latest wireless MCD (Figure 1). The former delivers microcurrent stimulation by connecting to an exogenous power source [11], and the wireless MCD does not need any accessories and just looks like a common woven dressing. The inherent electrical properties of the wireless MCD are activated by moisture from the wound through a pattern of alternately printed metallic dots, which employ the endogenous electric potential. The wireless MCD is commercially referred to as a bioelectric dressing (BED).

### Effects of microcurrent stimulation on wound healing

There have been many studies on the effects of microcurrent in both human and animal wound healing models [12–14] and on the cells involved in wound healing [15–17]. The application of the MCD, which transmits a low-level electric current to wounds, has been shown to facilitate different stages of wound repair process.

#### Anti-inflammation effects

The three phases of healing are inflammation, proliferation, and remodeling; they determine physiological wound healing. The normal process of healing in chronic wounds is interrupted by a prolonged inflammation phase [18, 19]. Both animal and human studies suggest that ES may aid in decreasing the duration of the inflammatory phase, thereby increasing the rate of healing, by reducing edema formation [20, 21] or reducing the pathogens in the wound area and decreasing their motility [22].

Using a rat ear skin model, Kaur *et al.* [20] studied the effects of microcurrent stimulation on the inflammation responses in intact skin. Tetradecanoyl phorbol acetate (TPA)-induced ear edema was performed, and the electric current was provided by galvanic zinc-copper (Zn-Cu) particles producing an electric current in the range of 70-90 μА (a current similar in magnitude to the endogenous wound current). By comparing the difference in ear weight and the levels of macrophage inflammatory protein 2 (MIP-2) between the control and Zn-Cu-treated mice, they found that Zn-Cu significantly reduced the TPA-induced edema response, thus inhibiting excessive inflammatory response.

In a rat hind limb model, Cook and colleagues [21] found that an applied microcurrent stimulated the movement of blue-dye-labeled albumin into the lymphatic vessels, increased oncotic pressure, and drew fluid into the vessels, thus reducing edema formation in the limb. Additionally, another study in rats that compared the therapeutic effects of microcurrent therapy and laser therapy in the process of wound healing reported that a microcurrent was effective in reducing the inflammatory reaction [23].

Similar to animal studies, anti-inflammation effects have been observed for electrical stimulation applied to human wounds. Lee *et al.* [24] used ultraviolet irradiation to induce inflammatory reactions in two areas in the lumbar region of 22 subjects. A microcurrent was applied to one region at an intensity of 50 μA. Measurements of the changes in chromatic red and luminance were taken over time, and a comparison of wound contraction in the two regions indicated that the applied-microcurrent region healed faster.

The antibacterial effects of ES also assist in reducing inflammation. All Gram-negative and Gram-positive microbes in the wound area carry a negative charge [25]. The positive polarity of the electric field attracts microbes and decreases their motility, thereby reducing the bacteria-caused inflammation response. In a study by Daeschlein *et al.* [22], the application of positive polarity had greater antibacterial effects than did negative polarity, regardless of whether the bacteria were Gram-negative or Gram-positive. In general, an applied microcurrent fosters an anti-inflammatory response and enhances wound recovery.

#### Effects of electrical stimulation on angiogenesis and blood circulation

The wound healing process consists of complex phases that begin right after injury, and there is an interaction between several tissues and cells [26]. The most crucial steps occur during the proliferative phase, ensuring the successful closure of the wound. In this phase, the formation of new blood vessels occurs through the bifurcation and extension of existing capillaries, an indispensable process for successful wound healing [27, 28]. ES has been demonstrated to induce important pre-angiogenic responses in in vitro mature endothelial cells.

Using an image analyzer, Zhao *et al.* [29] quantified the behavior of in vitro vascular endothelial cells before electric field (EF) exposure and after 8, 12 and 24 hours of EF (100 mV/mm) exposure. It showed that cells cultured without exposure to an EF had typical cobblestone morphology, with the long axis of each cell body oriented randomly. By contrast, endothelial cells cultured in an EF performed a dramatic reorientation, with their long axis lying perpendicular to the vector of the applied EF. This remarkable elongation and alignment in an applied EF resembles the angiogenic responses of endothelial cells. Additionally, angiogenesis is governed by endothelial migration. Zhao and colleagues [17] found that a small EF could also induce angiogenic responses in endothelial progenitor cells by causing significant directional migration and orientation. This was solely a response of the endothelial progenitor cells and did not require any other cell types.

In addition to promoting angiogenesis, microcurrent stimulation has been proven to increase the blood flow rate and promote local blood circulation. Park *et al.* [30] investigated the effect of microcurrent electrical stimulation, which was provided through a shoe, on blood circulation and pain in the feet of diabetes patients. The microcurrent delivered by the shoes was a pulsed microcurrent of no more than 300 μА. The subjects assumed a supine position before the measurement. The stable blood flow rate was then measured in that position, and a second measurement was taken after 1 h of walking exercise. The results showed that the increased blood flow rate of the experimental group was 1.19 mv/V, whereas that of the control group was 0.52 mv/V (*P* < 0.05). In this study, blood circulation in the feet of diabetes patients was significantly improved by applying microcurrent stimulation therapy. Similarly, Clarke *et al.* [31] reported that the lower extremity blood flow rate increased with applied of microcurrent stimulation in patients with chronic venous insufficiency.

#### Effects of electrical stimulation on granulation

During the proliferation phase of wound healing, granulation begins through increased collagen production [32]. Fibroblasts, which synthesize and secrete collagen protein, are the major component of granulation tissue and play an important role in the wound healing process. During the course of normal wound healing, fibroblasts at the wound edge are exposed to an electric field ranging from 40 to 200 mV/mm [2]. Various electric field conditions influence fibroblast migration, proliferation, and protein synthesis. Jennings *et al.* [33] explored the role of electric fields during the normal progression of healing, comparing gene expression in normal adult dermal fibroblasts exposed to a 100 mV/mm electric field for 1 h to non-stimulated controls. Significantly increased expression of 162 transcripts and decreased expression of 302 transcripts were detected using microarrays. Further, in a porcine model with 0.3 mm excisional wounds, direct current (50-300 μА) led to an increase in collagen synthesis from days 5 to 7, which was attributed to an augmentation of the number of collagen-producing cells. This increased number of cells could be due to proliferation or chemoattraction within the wound [34]. Electric fields appear to play an important role in controlling fibroblast activity in the process of wound healing.

Fibroblasts, the major cells of the dermis, show significant migration in the electric field, which is known as galvanotaxis. Sugimoto and colleagues [35] developed methods to measure galvanotaxis of fibroblasts and determined the optimal conditions for electrical stimulation. The results suggested that a low-intensity direct current promoted migration to the negative pole of human dermal fibroblasts. Tandon *et al.* [36] designed a wound-healing model in vitro for studying the effects of microcurrents generated by galvanic microparticles on cultured dermal fibroblasts. Single vertical scratches were made through the center of monolayers of human adult dermal fibroblasts. The imaging results suggested that the presence of galvanic microparticles significantly increased the speed of wound closing compared with the control. Taken together, the granulation phase is promoted by electrical stimulation through enhanced activity and migration of fibroblasts.

#### Effects of electrical stimulation on re-epithelialization

One key aspect of wound closure is re-epithelialization, namely the healing of the epidermis. Keratinocytes are the main cell population of the epidermis, and the migration and proliferation of keratinocytes are critical to re-epithelialization. Studies have found that keratinocytes migrated toward the negative pole and that applying direct-current electric field of as low as 10 mV/mm was enough to induce directional keratinocytes [37]. Banerjee *et al.* [38] studied the influence of BEDs on human keratinocyte cell migration. A scratch assay was performed, and cell migration was observed at 6 h and 9 h following the scratches. The results demonstrated with statistical significance that the gap closed faster in the presence of the BED than when treated with the placebo.

In addition, more findings from human and animal studies indicate accelerated wound healing rates and enhanced healing outcomes with the use of the BED [25, 39, 40]. In vivo porcine studies showed partial and full-thickness wounds treated with BED epithelialized significantly faster (up to 3 times at Day 5) compared to controls [40]. The interleukin-1α (IL-1α) response was reduced at Day 8, and the collagen markers, type-1 collagen (COL-1) and COL-3, evolved to perform a better long-term recovery in terms of remodeling and wound strength. The findings from a prospective case-series of skin graft harvest sites demonstrated the same results [25]. Thirteen patients who underwent skin grafting were enrolled. Half of the skin graft donor sites were treated with the BED and a semi-occlusive dressing (SOD), and the other half used only an SOD. The final results showed promise of faster healing, improved scarring, and improved patient subjective outcome with the use of the BED on acute wounds.

### Mechanisms of accelerated wound healing by ES

The exogenous application of microcurrent stimulation can direct cell migration and proliferation, stimulate angiogenesis, reduce the inflammatory response and improve wound healing. However, clinical application of the therapy remains elusive due to lack of a suitable device; hence, limitations in understanding the definite molecular mechanisms exist despite numerous completed studies. The main potential mechanisms to date are summarized below.

#### Increased expression of the BMP/SMAD signaling pathway

The expression of bone morphogenetic protein 6 (BMP6) has been proven in various tissues, including bone, skin and liver [41]. In skin, BMP6 signaling has been found to (1) regulate the developing and postnatal skin; (2) control cellular proliferation, differentiation, and tissue remodeling; and (3) govern a variety of pathological processes, including wound healing [42]. BMP6 is mainly produced by fibroblasts during wound healing, and it is found in the regenerating epidermis at the edge of the wound, as well as in fibroblasts of the granulation tissue. It is possible that the BMP6 is involved in the process of skin wound healing induced by microcurrent stimulation [36].

**Figure 2:**
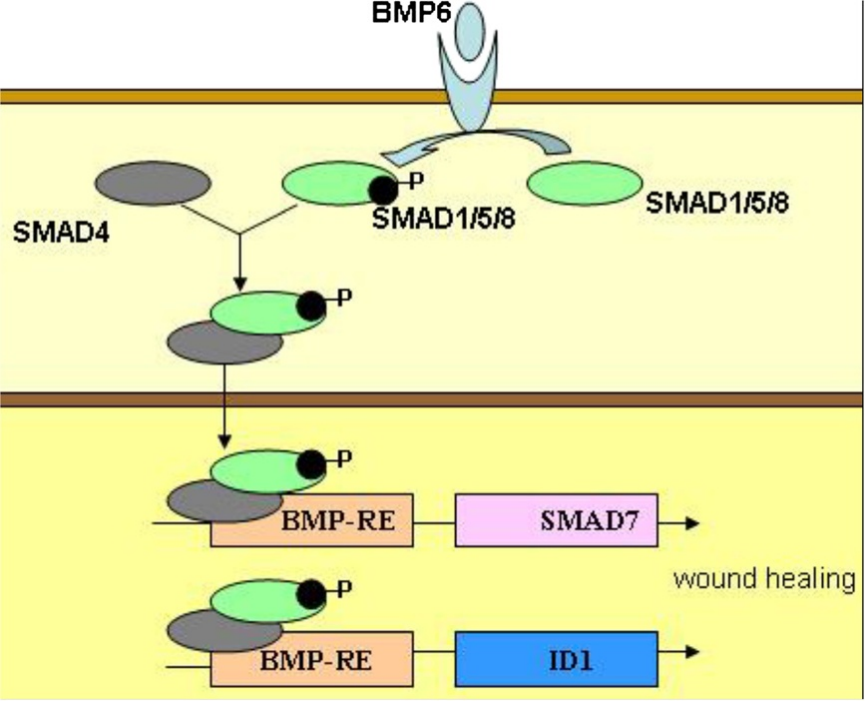
**The BMP/SMAD signaling pathway.** In the pathway, the interaction of BMP6 dimers with their receptors leads to the activation of the receptor kinase, followed by the phosphorylation of SMAD1/5/8 and the formation of the SMAD1/5/8-SMAD4 complex, which, when translocated to the nucleus, activates the promoters of the target genes and then regulates SMAD7 and ID1.

Tandon *et al.* [36] analyzed the response of fibroblast gene expression of BMP6 in response to the continuous application of microcurrent generated by galvanic microparticles. On days 1 and 3, significant increases in expression of the genes for BMP6, drosophila mothers against decapentaplegic protein 7 (SMAD7), and inhibitor of differentiation-1 (ID1) mRNA were observed. The up-regulation of BMP6, SMAD7, and ID1 is in accordance with the typical BMP signaling pathway (Figure 2). In the BMP/SMAD pathway, the interaction of BMP6 dimers with their receptors leads to the activation of the receptor kinase, followed by the phosphorylation of SMAD1/5/8 and the formation of the SMAD1/5/8-SMAD4 complex. The complex translocates to the nucleus, activating the promoters of the target genes [43] and then regulating SMAD7 and ID1 [44, 45]. A proposed representation of the BMP6 signaling pathway could be either *via* ID1, stimulating proliferation and cell motility after skin injury [46], or *via* SMAD7, which stimulates wound healing [47]. The potential mechanism to stimulate BMP6 into action may relate to the electrons produced by microcurrent stimulation. Studies have shown that the electrons from iron in the liver may influence the BMP6 signaling pathway [43], although this effect has not been reported in the skin. Thus, it is possible that microcurrent stimulation mimics these effects in skin by producing free electrons, thereby activating the BMP/SMAD signaling pathway.

#### Suppression of NF-κB activity

Nuclear factor-κB (NF-κB) is a nuclear transcription factor and a key molecular target because it modulates many types of genes governing immune and inflammatory responses [48]. In most cells, NF-κB proteins are separated in the cytoplasm, bound by a member of the NF-κB (IκB)-inhibitor family, which include IκBα, IκBβ, and IκBϵ [49]. The exposure of cells to extracellular stimuli, such as tumor necrosis factors (TNF), leads to the activation of the IκB kinase (IKK) complex, which includes two catalytic subunits, IKKα and IKKβ, and results in the phosphorylation and ubiquitination of the IκB proteins and their proteasome mediated degradation [50]. When IκB is degraded, NF-κB subunits, including P65, P50 and relA, are free to transfer to the nucleus and turn on genetic transcription of downstream targets. Expression of the pro-inflammatory cytokines, such as IL-1α, IL-2, nitric oxide (NO) and TNF-α, is transcriptionally regulated by NF-κB [51].

In an attempt to clarify the potential mechanism of anti-inflammation reaction by low-level ES, Kaur and colleagues [20] treated the cultured primary keratinocytes with Zn-Cu galvanic particles, followed by treatment with TNF-α for 24 h, and then examined the activity of NF-κB. The activity of TNF-α induced NF-κB was almost entirely inhibited. They followed the degradation of IκBα in the untreated and Zn-Cu treated keratinocytes. TNF-α treatment caused the degradation of IκBα and P65 phosphorylation [52], while Zn-Cu treatment inhibited this effect. And further, the result was confirmed by visualizing the nuclear localization of the NF-κB P65 subunit. The P65 subunit translocated to the nucleus with the treatment of TNF-α, whereas cells pretreated with Zn-Cu for 2 h followed by TNF-α stopped NF-κB from translocating. In summary, suppression of NF-κB activity is one of the potential pathways through which microcurrent exerts its anti-inflammatory effects.

#### Activation of PI3K signaling pathway

Under the effect of chemoattraction, cells polarize and migrate directionally, and the activation of phosphoinositide3-kinases (PI3K) is evident on the leading edge; meanwhile, phosphatase and tensin homolog (PTEN), the negatively regulating PI3K factor, collect at the opposite pole of the cells [53]. Thus PI3 kinase and PTEN are called ‘compass molecules’ for directional sensing and polarization in chemotactic cells [54].

Studies have found that the physiological electric field activated the PI3K-signaling pathway in neutrophils and keratinocytes cultured in a serum-free medium [55]. When the cells that expressed green fluorescent protein-marked Akt (Akt-GFP) were exposed to an electric field, Akt-GFP dispersed and polarized in the cell migration direction; this indicated that PI3K was activated in a polarized manner at the cathode-facing side and that cells extended to form the leading edge [56]. Once the polarity of the electric field changed, Akt-GFP oriented to the new cathode-facing side; this outcome shows that the electric field can initiate the directional activation of PI3K in the cells. At the side of the cell where PI3K was activated, the cell membrane stretched out, and the cell showed migration in that direction [55, 57]. Moreover, using mutant mice whose PI3K was genetically knocked down, the important function of PI3K in modulating the electric field-induced directional cell migration was proven [55]. This characteristic abated electrotactic migration in wound healing in cell and tissue cultures. In cellular experiments, tissue-specific removal of the PTEN gene in keratinocytes intensified Akt phosphorylation; this mutation in turn enhanced electrotaxis. Thus, PI3K and PTEN represent a class of molecules modulating electrotaxis that have been confirmed at gene levels.

In addition, electric fields of physiological magnitude also activate other signaling pathways, such as the epidermal growth factor receptor (EGFR) and mitogen-activated protein kinases (MAPK) [58]; the activation of these signaling pathways is associated with a direction of cell migration.

#### Enhancement of VEGF release

Angiogenesis is an important event that is involved in the healing of various types of wounds and is primarily modulated by the release of vascular endothelial growth factor (VEGF) from endothelial cells, platelets, keratinocytes, and fibroblasts [59]. VEGF regulates the multiple biological functions of endothelial cells, thereby enhancing the production of vasodilatory mediators, increasing vascular permeability, and stimulating their migration, proliferation, and formation [27]. As the basic angiogenesis regulator, the VEGF improves angiogenesis and plays a crucial role in the healing of wounds. Microcurrent stimulation has been demonstrated to promote angiogenesis, a mechanism related to the enhancement of VEGF release.

Experimental studies on endothelial cells have shown that ES significantly increases the levels of VEGF release. Bai and colleagues [60] cultured endothelial cells of a human umbilical vein in a 200 mV/mm direct-current field and then quantified the release of VEGF. A marked increase of VEGF in the culture medium was observed as early as 30 min after the exposure to the electric field. The level decreased between 1 and 2 h, and rose again at 4 h, reaching the highest level by 24 h. Additionally, the expression of VEGF mRNA demonstrated significant up-regulation at 4-24 h. In rat models, Asadi *et al.* [61] proved that a direct current of 600 μА was more effective than a monophasic pulsed current of 2.5-3.0 mA in promoting wound healing of a full-thickness skin incision due to higher skin VEGF levels on the 7th day.

Although it is known that the application of microcurrent stimulation promotes the release of VEGF both in vitro and in animal models, it is encouraging that the same results are observed in clinical practice. Ferroni *et al.* [62] conducted a pilot study to verify the effect of ES on circulating VEGF levels in patients with peripheral arterial ulcers. Nine patients were recruited and received local pulse ES (100 μА max). Samples of peripheral venous blood were withdrawn, and the VEGF levels were detected in samples obtained before, during, and after treatment. An immediate increase in the mean VEGF level was observed during the treatment, and a peak appeared at 7 min. Sebastian *et al.* [63] compared cutaneous wound healing in healthy human volunteers with or without an electric ES. A punch biopsy of full-thickness skin was obtained and then treated with ES. VEGF was assessed and showed significant expression in the treatment group (66%) and the non-treatment group (38%), compared with 24% in normal skin at the 14th day. These data indicate the promising effect of microcurrent for enhancing the release of VEGF and promote wound healing.

#### Improvement of mitochondrial function

Wound healing is a complex process that includes the proliferation and differentiation of different types of cells and thus requires a higher energy supply [64]. Mitochondria are important intra-cellular organelle in eukaryotic cells and are the main venues of oxidative phosphorylation and adenosine triphosphate (ATP). Thus, the production of ATP benefits from improved mitochondria and accelerates skin wound healing.

It is proposed that external electrical stimulation may improve mitochondrial function by generating moderate superoxide radicals in cultured human keratinocytes exposed to low intensity electric field [38]. A superoxide radical works as an electron donor for oxidative phosphorylation [65]. Thus, it is expected that improved performance of the tricarboxylic acid (TCA) cycle produces more nicotinamide adenine dinucleotide hydrogen (NADH) and flavin adenine dinucleotide hydrogen 2 (FADH2), which then enters the electron transport chain, contributing to increased mitochondrial membrane potential and improved mitochondrial function. Using fluorescent dyes JC-1 and tetramethylrhodamine methyl ester (TMRM) to measure mitochondrial membrane potential, Banerjee and colleagues [38] found that the treatment of keratinocytes with a bioelectric dressing for 24 h showed significantly high red fluorescence with both JC-1 and TMRM; this result represents a higher mitochondrial membrane potential.

Further, as a potential consequence of a hyper-active TCA cycle, the pool of pyruvate, which is the primary substrate for TCA cycle, is exhausted; this results in an increased rate of glycolysis to refill the pyruvate pool [66] and an increased rate of glucose uptake. This phenomenon was demonstrated in HaCaT cells treated with a bioelectric dressing for 24 h compared with the control. The direct evidence of microcurrent stimulation inducing ATP production was conducted in tissue cultures [67]. The cultured skin tissue of male Wistar rats, just finishing their first hair-cycle at 21 days of age, was electrically stimulated with different levels of microcurrent for 2 h. ATP concentrations in controls and electrostimulated skin samples were assayed by the luciferin-luciferase reaction. With electric currents ranging from 10 to 1,000 μА, this experiment showed that the ATP levels in the tissue were increased to three to five times the untreated control levels; with currents exceeding 1,000 μА, the ATP concentrations leveled and were even lower with higher currents. Overall, microcurrent stimulation promotes mitochondrial function, induces more ATP synthesis, and ultimately accelerates wound healing. Other wound healing mechanisms related to ion channels of Ca^2+^ and Na^+^ have been proposed but are not well known.

## Conclusions

Because it is difficult to imitate wound healing in vivo, general understanding of the healing mechanisms with applied low-level electric currents has progressed slowly. Although microcurrent stimulation for accelerating wound healing has been studied for several decades and various types of ES devices have been applied in clinical practice, many questions remain about the underlying mechanisms and the intensity and time at which stimulation should be applied to achieve the best effect. These studies supporting the positive effect of electrical stimulation by MCD in the acceleration of wound healing suggest that further research is justified and necessary.

Although a large number of advances in the basic research of wound repair have been made, the rate of transformation from theory to practical application has been slow. One reason is the lack of an accurate wound model and evaluation system, the other is the deficiency in the technological innovation of these medical devices. For example, MCD devices should be more effective, comfortable, and portable; the design of MCDs should constantly be evolving; and new MCDs should be assessed. Additionally, the creation of a standard protocol to measure wound-healing parameters would provide standardized data and facilitate future analyses. What we can do now is integrate various effective therapies into one comfortable and portable wound care device; the advancement of MCDs is just the beginning. We hope that cutting-edge interdisciplinary research will result in new dressing devices and therapies that could be put into clinical practice.

## Abbreviations

Akt-GFP: Green fluorescence protein marked Akt
ATP: Adenosine triphosphate
BMP: Bone morphogenetic protein
COL-1: Type-1 collagen
EGFR: Epidermal growth factor receptor
ES: Electric stimulation
FADH2: Flavin adenine dinucleotide hydrogen 2
ID-1: Inhibitor of differentiation-1
IL-1α: Interleukin-1α
IκB: Inhibitor of NF-κB
IKK: IκB kinase
MAPK: Mitogen-activated protein kinase
MCD: Microcurrent dressing
MIP: Macrophage inflammatory protein
NADH: Nicotinamide adenine dinucleotide hydrogen
NF-κB: Nuclear factor-κB
NO: Nitric oxide
PI3K: Phosphoinositide3-kinases
PTEN: Phosphatase and tensin homolog
SMAD7: Drosophila mothers against decapentaplegic protein 7
SOD: Semi-occlusive dressing
TCA: Tricarboxylic acid
TMRM: Tetramethylrhodamine methyl ester
TNF: Tumor necrosis factor
TPA: Tetradecanoyl phorbol acetate
VEGF: Vascular endothelial growth factor.
